# Invisible but palpable– gender norms in childbirth: A focused ethnography on woman-midwife interaction and birth practices in two Swedish hospital labour wards

**DOI:** 10.1186/s12884-025-07554-8

**Published:** 2025-04-10

**Authors:** Agneta Westergren, Kerstin Edin, Bo Nilsson, Monica Christianson

**Affiliations:** 1 Sexual and Reproductive Health, Department of Nursing, Umeå University, Umeå, SE-901 87 Sweden; 2 Department of Epidemiology and Global Health, Umeå University, Umeå, Sweden; 3 Department of Culture and Media Studies, Umeå University, Umeå, Sweden

**Keywords:** Birth setting, Childbirth, Focused ethnography, Gender roles, Hierarchy, Intrapartum interventions, Medicalisation, Midwifery, Physiological birth, Power relations

## Abstract

**Background:**

Swedish labour care is becoming increasingly medicalised, with rising rates of intrapartum interventions such as induction and augmentation of labour, epidural analgesia, and caesarean section. This study aimed to explore the paradox of the increasing medicalisation of childbirth despite the vast evidence of the benefits of low-intervention physiological birth.

**Methods:**

Focused ethnography was used to study woman-midwife interactions during labour and birth and the everyday practices of midwives in two Swedish labour wards. After birth, the women and midwives were interviewed. Thematic analysis was used to analyse the data, and the study design and interpretation of results were informed by a social constructionist view of gender.

**Results:**

The analysis resulted in three themes, mirroring the pillars on which labour and birth care rests– the labour care organisation, the midwives, and the women who give birth. The organisation was hierarchical and based on traditional masculine values such as rationality, efficiency, and productivity. The midwives tried to balance the needs of the birthing women and the organisational demands of throughput. As action and technological skills are more noticeable and linked to masculinity, and thus more valued than the invisible feminine-coded emotional care work of supporting a woman in labour, the midwives became task-oriented and more focused on ‘doing’ than on ‘being’. This led to more birth interventions, less support for the birthing women, and to occupational stress and stress of conscience for the midwives. Normative expressions of femininity were observed in the birthing women, such as placing the needs of others before their own and acts of compliance, which sometimes led to unconsented interventions.

**Conclusions:**

We suggest that societal gender norms and gender-based hierarchies in combination with modern society becoming progressively risk-laden and technology-oriented, have contributed to an increasingly medicalised and interventionist labour and birth care organisation, where physiological birth is rare. Awareness of how gender norms inform labour and birth care practice may be one way to make visible and to recognise all aspects of midwifery care, as well as help flatten hospital hierarchies, improve working conditions for midwives, promote physiological birth, and limit unnecessary and unconsented interventions for the birthing women.

## Background

Globally, there are two extremes on the continuum of maternal health care: too little, too late and too much, too soon. While the first describes a lack of resources and unavailable or withheld care, the second one describes a trend towards excessive, unnecessary, or inappropriate use of medical interventions during childbirth, i.e. an over-medicalisation of normal pregnancy and birth [1]. In both cases, the lives and well-being of pregnant and birthing women and their babies are at risk.

In many middle and high-income countries, maternal health care has come to involve several practices designed to ‘initiate, accelerate, terminate, regulate, or monitor the physiological process of labour [2]. While medical interventions are sometimes vital for a safe birth, the overuse of interventions and the reliance on technology may at times increase risks for women and their babies, rather than mitigating them [2–5].

An increasing body of evidence demonstrates both physical and psychological benefits of a physiological birth with as little disturbance and intervention in the process as possible [6, 7]. Promoting, supporting, and protecting the innate hormonal physiology of women and babies optimises labour and birth, newborn transition, breastfeeding, maternal adaptation, and maternal-infant attachment [8]. A vital component in facilitating physiological birth is woman-centred midwifery care, also known as the social model of care, recognising that childbirth is more than the delivery of a healthy baby and more often than not a natural, physiological event [9]. Midwifery care has been found to reduce maternal and neonatal mortality and morbidity, reduce stillbirths and preterm birth, decrease the number of unnecessary interventions, improve maternal satisfaction with care, and improve psychosocial and public-health outcomes [10–13].

Despite the many advantages of woman-centred care following evidence-based guidelines that recommend a physiological approach to labour and birth, on the contrary, a risk-based and interventionist approach informs practice in obstetric units [2, 14]. This approach is described as the medical model of care, built on the assumption that childbirth requires medical control and surveillance to keep birth safe, and that childbirth is ‘normal only in retrospect’ [9].

In line with the International Confederation of Midwives, the Swedish Association of Midwives emphasize the promotion of normal, physiological birth as scope of practice for the profession [15, 16]. Midwives in Sweden work independently as primary care providers for pregnant and birthing women, and in complicated cases, they work in collaboration with obstetricians. Yet, despite midwives’ professional mandate to promote and facilitate undisturbed, physiological birth, intrapartum interventions are rising [17, 18].

Previous research has raised the concern that modern labour and birth care has undermined midwives’ knowledge of how to support the physiological and emotional process of birth without routine intervention [3, 19, 20]. The aim of this study was, through a gender perspective, to explore the paradox of childbirth becoming increasingly medicalised despite the vast evidence of the benefits of low-intervention physiological birth. The concept of social construction of gender and gender-based hierarchies informed the design, analysis, and interpretation of the results, which to our knowledge has not been done before in the context of Swedish midwifery research.

## Methods

### Gender as a social construct

The theoretical framework guiding this study is the social constructionist view on gender, positing that gender is a social construct that varies with time and place and is something one inadvertently does, not is [21, 22]. While a person’s biological sex refers to physical and biological features like sex organs, chromosomes, or hormones, gender describes the culturally and socially constructed norms, roles, and entitlements associated with these biological differences [23]. Simone de Beauvoir famously claimed that ‘one is not born, but rather becomes, a woman’, arguing that behavioural traits associated with women and men are culturally learned or acquired rather than innate [24]. From an early age, girls and boys are socialised into conforming to certain norms and expectations based on their biological sex, from which follows the development of a gender identity in line with societal norms of femininity and masculinity. These gender identities may present individuals with benefits and possibilities on the one hand and/or injustices and disadvantages on the other [21].

The feminist critique of gender constructs is that they create a hierarchy, and thus a power relation, in which one takes precedence over the other [23, 25]. Most gender systems are patriarchal and ascribe greater value to men and traits associated with masculinity than to women and traits associated to femininity [23]. A bit simplistic, traits associated with masculinity, and generally more valued in Western societies, are strength, reason, and action, while feminine traits are described as weakness, emotion, and passivity [25, 26].

With women giving birth, and with 99.7% of the midwives and 77% of obstetricians/gynaecologists in Sweden being women, childbirth is a heavily female domain [27]. Exploring childbirth through a gender constructionist lens may shed light on how societal and cultural gender constructions and norms, and the power relations that follow, affect the labour care organisation, the midwives, and ultimately the women who give birth.

### Focused ethnography

Healthcare professionals may not always be able to describe how they practice their profession, and there may be discrepancies between what people say in an interview and what they do in practice [28]. While traditional ethnography aims to gain an understanding of the behaviours of a social or cultural group, focused ethnography has emerged as a method to understand a distinct issue or shared meanings and practices in a specific, often medical, setting or sub-culture [29]. As in traditional ethnography, this method is also based (mainly, but not solely) on participant observation, but due to the often narrow scope of the study and time limitations, focused ethnography allows for a more pragmatic and rapid data collection, where the researcher may or may not be familiar with the setting [30]. In this study, focused ethnography in the form of participant observation during labour and birth and subsequent interviews with the birthing women and the midwives, was used to capture the discourses, interactions, and practices surrounding childbirth in two labour and birth units.

### Approaching the field

A common challenge in ethnography is gaining access to the field, which often involves being accepted by specific gatekeepers [31]. Initially, two gatekeepers (heads of departments at two hospitals) declined participation in the study, whereas two other hospitals were approached. The heads of departments on these sites welcomed the study, and before commencing the fieldwork, the first author, AW, visited both sites and informed the staff about the aim and procedure of the study.

### Settings

The two hospital labour and birth units (Site I and Site II), both with an annual rate below 1,000 births, were in different healthcare regions, and provided postnatal and gynaecological care in the same facilities. Most of the midwives, along with assistant nurses, therefore covered all specialties, working one day with birthing women and the next with new families, or with women with various gynaecological conditions. With an average rate of approximately two births per day at both sites, one-to-one care was often but not always possible. Obstetricians, gynaecologists, and/or resident and intern physicians were seen on the wards mainly during rounds, and neonatologists or paediatricians were available in the hospitals during daytime. When on call during the night, doctors who lived close to the hospitals were in their homes. Anaesthesiologists were often available at short notice, although sometimes hindered by responsibilities on other wards.

At one site, active management of labour was part of routine care: women’s cervixes were expected to dilate by one centimetre per hour, otherwise contractions would be enhanced primarily through amniotomy, and if that did not suffice, through an intravenous drip of synthetic oxytocin. During the study period, there were plans to introduce this method at the other site as well. At both sites, a partogram was used to monitor labour progress. Primarily obstetricians developed policies and guidelines for clinical practice without the involvement of midwives.

### Recruitment and participants

With the intent to observe vaginal birth and to interview the women and midwives afterwards, women with planned caesarean section were excluded, as were women who did not speak Swedish or English. To capture a variety of experiences, all other women were included, regardless of age, parity, ethnicity, length of pregnancy, onset of labour, or preferences for pain relief.

When a woman came to the labour ward and was considered suitable for the study by the midwife who was to assist her, informed and written consent was first obtained from the midwife, who had been previously informed of the study. None of the midwives in question declined participation. The midwife then asked the woman if she would consider having a researcher, who is also a midwife (AW), present during labour and birth and to be interviewed about her experiences afterwards. All women who were asked accepted. Once they had agreed, AW entered the room and presented herself and the study to the woman and her partner and provided written information about the project. The couple were left alone for a while to read the information and then the woman was asked to sign a written consent form, stating that participation was optional and that she had the right to rescind consent at any time without any impact on the care she would receive. Once the consent form was signed, participant observation followed until the baby was born, between four and ten hours. Besides the participants (midwives and women), the women’s partners, and the researcher, there was an assistant nurse present during the pushing phase in all births, a senior midwife colleague present during three births, one physician present in one birth, and two physicians present in another.

Childbirth is difficult to plan, and there were not many babies born during the fieldwork, there were also some cases where the midwife in charge made the decision to not involve a woman in the study, mainly due to psychosocial issues or language barriers. There were also a few quick births that took place where the staff did not find the time to call the researcher, who was on constant call when not on site.

### Data collection

To promote methodological rigour and ensure consistency across sites and participants, the research group designed an observation guide. The guide focused on the interaction between the midwife and the birthing woman, such as use of verbal and non-verbal language, the woman’s and the midwife’s strategies for handling labour pain, as well as hospital routines and intrapartum interventions. The physical environment of the birth room, such as interior design, access to showers, bathtubs, birth tools, etc., was also considered. To not disturb the sensitive process of giving birth, AW avoided taking notes in the birth room. Shadowing the midwives, who continuously went in and out of the birth room to check on other patients or to assist colleagues, AW used the moments spent outside the birth room to fill in the observation guide and to write down thoughts, interpretations, and reflections in the form of analytical memos to be either further explored or discarded. The observation guide and memos were later used to support recollection and integrated into the more extensive field notes which AW wrote down in private, directly after each observation, covering about 20 pages.

Two interview guides were also designed by the research group, one for the women and one for the midwives, focusing on their perceptions of pain and pain relief, support during labour, and balance of power in the birth room. The interviews were recorded and with the midwives they lasted on average 47 min, while those with the women lasted on average 22 min. All interviews were conducted on-site, in a private room, one or two days after birth.

Collected data thus included the documentation of AW’s observations in the form of memos and field notes, along with drawings and photos of the labour ward and formal interviews with the observed women and midwives. All data collection was done by AW, who aimed to observe at least five births in each setting and spent ten days (covering all shifts) on each site.

### Reflexivity

Qualitative (and feminist) research acknowledges and stresses the active role of the researcher in the development of knowledge [29]. Reflexivity helps the ethnographer to be aware of how the self, i.e. background, personal opinions, and past experiences, may influence study design, data collection, and theoretical interpretations [30]. Before joining academia, AW was a practising midwife, albeit in a different clinic from those under study. Questions thus arose on whether AW’s position as a researcher would be confused or conflicted with her role as a professional midwife. Considering that having the insider perspective as a midwife had its advantages– being familiar with the setting, the jargon, and the procedures in the birth room– AW decided to embrace the multiple identities and introduce herself both to staff, women, and their partners as a researching midwife.

Considering the ethical aspect of observing women during labour and birth, AW decided to dress in scrubs to make the birthing woman feel more comfortable and less observed to avoid slowing down or inhibiting labour progress. Emphasising the collegial role also mitigated the observed midwives’ potential feelings of being under scrutiny. During the observations, AW tried to keep a low, but accommodating and courteous profile, assisting both midwife and woman if desired, partaking in conversations, but being mindful of not overstepping boundaries and interfering in the woman-midwife interaction.

Although a researcher being familiar with the field under study can allow the exploration of certain phenomena that may go undetected by an outsider, there are potential drawbacks, i.e. being so accustomed to the context that some practices are taken for granted or as inevitable. To meet the researcher subjectivity requirements, the observation and interview guides previously constructed together with co-authors MC and KE were followed. Apart from the actual data collection, all co-authors participated in every step of the research process and had access to fieldnotes and interview transcripts.

### Data analysis

The interviews were transcribed verbatim and the data was analysed following Braun and Clarke’s [32] six steps of thematic analysis. Initial repeated reading of both transcripts and field notes was undertaken and initial codes were noted in the document’s margin. The codes were gathered and a search for patterns and preliminary themes followed. Through an iterative process, going back and forth between transcripts, field notes, codes, and preliminary themes, the analysis resulted in three major themes with three subthemes respectively. The results were continuously discussed in the research group until consensus was reached.

For enhanced credibility, researcher triangulation was used, i.e. involving researchers knowledgeable in midwifery science, gender studies, public health, and ethnology. Quotes from the participants were also included to emphasise their voices and perspectives. Transferability was met by providing a thick description of the settings. Dependability was addressed through keeping a systematic record of fieldnotes, reflexive notes, and interview transcripts, clearly documenting the research process, and involving the research team in every step of the process. Confirmability was achieved by being open about theoretical, methodological, and analytical choices throughout the study as to ensure that the findings are derived from the data.

All observed births are represented in the result, either through quotes from the interviews or in extracts from the field notes from the observations. For reasons of anonymity, the quotes and the extracts are randomly numbered, and do not connect midwives, women, or observations.

## Results

Eight midwives and eight women accepted participation, five in one hospital and three in the other. The average age of the midwives was 39.6 years, they were all women and Swedish speaking, and their work experience as a midwife ranged from two months to almost 30 years. Background information on the birthing women and some details about their births are shown in Table 1.

**Table 1:** Maternal characteristics, intrapartum interventions, self-reported pain and birth experience (*n* = 8)

MATERNAL CHARACTERISTICS	Number
Age (years)	23–38
Swedish speaking/English speaking	7/1
Higher/secondary education	3/5
Primipara/multipara	5/3
Length of pregnancy (gestational weeks)	37–41
Partner present	8
**INTRAPARTUM INTERVENTIONS**	
Induction of labour	3
IV-line	8
Nitrous oxide	8
Epidural analgesia	4
Pudendal block	2
Amniotomy	3
Augmented labour	6
Continuous fetal monitoring	7
Intermittent urinary catheterisation	6
Use of birth tools^1^	5
Birth positions^2^	8
Directed pushing	5
Hands-on perineal protection	8
Early cord clamping^3^	4
Active management of third stage^4^	8
Sutured vaginal laceration	6
**SELF-REPORTED PAIN AND OVERALL BIRTH EXPERIENCE**	
VAS Pain^5^	8.3
VAS Overall birth experience^6^	8.1

^1^Woman being encouraged to try alternate positions with walker, birthing ball, or birthing stool

^2^Four in lithotomy position, two in supine position, two semi-sitting in bed

^3^Within 60 s of birth

^4^Prophylactic uterotonic, early cord clamping, controlled cord traction

^5^Visual Analogue Scale, rated by the women after birth, 0 = No pain and 10 = Worst possible pain

^6^Visual Analogue Scale, rated by the women after birth, 0 = Very bad and 10 = Very good

The analysis resulted in three major themes mirroring the pillars on which labour and birth care rests– the labour care organisation, the midwives, and the women who give birth: *A hierarchical organisation based on risk*,* efficiency*,* and productivity; Midwives in between ideals and reality;* and *Birthing women trusting in the system* (Table 2).

**Table 2:** Main themes and subthemes

A hierarchical organisation based on risk, efficiency, and productivity	Midwives in between ideals and reality	Birthing women trusting in the system
A risk-focused high-tech birth environment	The emotional labour of supporting women in labour	Placing the needs of others before their own
Inadequate staffing leading to more birth interventions	Multitasking midwives balancing birthing women’s needs and organisational demands	Compliance presupposed
Hospital hierarchies and power dynamics	Midwifery at risk of becoming a lost art	Unconsented interventions and self-blame

### A hierarchical organisation based on risk, efficiency, and productivity

The way labour care was organised, and the way the birth rooms were designed, indicated that it was built on the assumption of childbirth as a risky and medical event and at the same time efficiency driven and focused on patient flow. Inadequate staffing was counterbalanced through various intrapartum interventions, such as iv-lines, epidurals, and continuous fetal monitoring, which allowed for a more efficient use of midwives. The midwives viewed the organisation as hierarchical, with physicians formally in charge of care, at times leading to disagreements on adequate care and subsequently to interprofessional tension.

#### A risk-focused high-tech birth environment

The two labour and birth units were both located in hospitals, which may have influenced their architecture and interior design.

*‘The wards on both sites were reminiscent of a standard hospital ward: a long corridor with closed doors leading to birth rooms on both sides. The midwives’ station was in the centre of the ward*,* equipped with computers*,* books*,* forms*,* papers*,* folders containing unit policies*,* a whiteboard showing birth room number*,* each labouring woman’s name*,* parity*,* gestation week*,* risk assessment*,* and birth-related information (amniotomy*,* epidural analgesia*,* group B strep*,* etc.)*,* and a monitor where the cardiotocographs of all labouring women were displayed. The patient alarm system had a shrill sound and a red blinking light*,* audible and visible in every room on the ward. Both sites had a neonatal resuscitation room on the premises. The operating room to be used in case of caesarean section was in another part of the hospital. Showers were available on both sites*,* and one had a large bathtub. Waterbirths were not allowed.’* ~Observations from Sites I and II~.

On both sites, the birth rooms were designed from a perspective of medical functionality and hygiene. Overall, the rooms forwarded a view of birth as risky and in need of various technological tools to keep it safe.

*‘On Site I*,* the birth bed was placed around a corner so if a woman were lying on it*,* she could not see who entered the room. Assuming the woman is lying on her back*,* her lower body would be exposed to anyone entering the room. On Site II*,* the bed was placed directly across from the door*,* with the woman’s lower body on full display if she was sitting or lying on the bed. Moreover*,* the beds were placed in the centre of the room and surrounded by medical equipment such as electronic fetal monitors*,* intravenous drip stands with infusion pumps*,* tubes for nitrous oxide (hidden behind sliding doors on one site)*,* and a bright surgical light was positioned over the bed. White*,* ironed bed linen*,* along with smooth*,* shiny*,* easy-to-clean counters and floors*,* enhanced the impression of birth as a clinical*,* hygienic event. Birth tools such as walkers and birthing balls were available but not kept in the birth rooms and were therefore not visible to the women to encourage them to move around and be active in birth.’* ~Observations from Sites I and II~.

On one site there were three monitors in the birth room, one behind the bed, one beside the bed, and one in front of the bed, allowing both the midwife and the couple to see the cardiotocography (CTG) displayed from every angle in the rooms. Apart from giving the impression of birth as risky and high-tech, it also at times contributed to added stress in the room.

*‘The birth room*,* with its lack of colours and textiles gives a cold and sterile impression*,* and there is a slight echo in there. The midwife is focused on the electronic fetal monitor*,* continuously monitoring the fetal heart rate and the uterine contractions. The monitor’s sound is audible and hard*,* like someone bouncing a slightly heavier than normal table tennis ball at a rate of on average 150 bounces per minute and doing so for three hours straight. The ping-pong sound reverberates in the room*,* and when the fetal heart rate drops during contractions*,* both the midwife and the birthing woman and her partner anxiously fix their eyes on the monitor.’* ~Observation 8~.

#### Inadequate staffing leading to more birth interventions

Constant understaffing and not knowing until the beginning of the work shift what unit they would be placed to work in– labour, postnatal, or gynaecological, left especially junior midwives feeling apprehensive and insecure with persistent feelings of inadequacy and bad conscience. Although one-to-one care was mostly possible, the midwives often felt their efforts were insufficient and expressed frustration with the way their work was organised.

*‘It would be great to be assigned only one woman in active labour*,* not several*,* but it doesn’t work that way. But it’s really frustrating to care for a woman and feel that you don’t want to leave her* [but to have to].*’* ~Midwife 6~.

To manage the sometimes-overwhelming workload, the midwives used various interventions to facilitate workflow and trying to save time. For instance, all women had iv-lines, all but two women had their labours augmented and were catheterised instead of encouraged to go to the bathroom, and half of them had an epidural (Table 1). One midwife expressed that suggesting an epidural to the women made their job easier, as the epidural reduced the woman’s need for time-consuming continuous support from the midwife.

*‘The epidural*,* sure*,* it’s good for the women*,* too*,* and they often say*,* “I wouldn’t have made it without it”*,* but I ask myself: what would we* [the midwives] *do without it?’* ~Midwife 3~.

Continuous CTG made it possible for midwives to monitor several women simultaneously without having to be present in the birth room, thus enabling more efficient use of midwives to the benefit of the organisation. However, this risked overshadowing the birthing woman’s needs for support and contributed to split attention and added stress for the midwife. Moreover, with the woman on the CTG, the midwives (and partners) often placed their focus on the monitor displaying the woman’s contractions and the fetal heart rate, rather than on the woman.

*‘Her labour is very intense. She is breathing nitrous oxide*,* but not finding the right technique*,* standing on the tips of her toes beside the birth bed*,* writhing*,* squirming*,* and screaming from the labour pain. The midwife focuses on retrieving the woman’s electronic medical record on the computer and spends much time at the computer*,* documenting care*,* or looking at the fetal monitor. She is also caring for another woman in labour*,* and through EFM she can surveil that woman’s labour on the computer*,* being able to monitor both women at the same time. With her back turned to the woman*,* she now and then says encouraging words from a distance.* ~Observation 4~.

#### Hospital hierarchies and power dynamics

Although the midwives were the primary care providers for the labouring and birthing women, only involving obstetricians in case of a birth complication or in caring for a high-risk patient, they were not involved in developing guidelines regulating their work. To the midwives’ dismay, this was a task for the obstetricians. There were observations where the hospital hierarchy and physician prerogative were apparent, for instance in case of disagreements on adequate care. Rather than standing up for their professional expertise, the midwives kept quiet and became subservient, thus contributing to upholding hospital hierarchies and power imbalances. Ultimately, the silence of the midwives risked negatively affecting the birthing women.

*‘The woman’s labour has been induced and is progressing quickly. The midwife wants to lower the speed of the synthetic oxytocin drip because of abnormalities in the fetal heart rate*,* but the resident physician tells her to continue raising it and subsequently performs a vacuum extraction*,* forcefully pulling the baby out. Despite the abnormal fetal heart rate*,* the baby is fine and cries instantly.’ ~*Observation 1*~*.

Commenting on this birth in the subsequent interview, the midwife stated that she would have managed the birth differently, had she been in charge.

*‘Had it been up to me*,* I would have terminated the drip once she was in active labour to give her body a chance to produce endogen oxytocin. I understand that the induction of labour was necessary and that the baby needed to be born*,* but I think that one could have tried to lower or completely terminate the drip… I don’t think this doctor is very experienced in delivering babies… I wouldn’t have chosen vacuum extraction*,* the woman could have breathed that baby out*,* but at the same time*,* I feel that the doctor needed the practice* [of a vacuum delivery].*’ ~*Midwife 8*~*.

Interprofessional tensions were also apparent in the way some of the midwives expressed that the obstetricians encroached on their territory of normal birth, while not always following best practice, discussed in terms of birthing position:

*‘The struggle continues*,* now there’s a discussion about the birth stool. Many women want to give birth on the birth stool*,* but the doctors are afraid of severe tearing*,* so they have forbidden it. So*,* women are placed on their backs*,* preferably with their legs in leg rests*,* so we have a full view of the pelvic area.*’ ~Midwife 3~.

### Midwives in between ideals and reality

When interviewed, the midwives valorised physiological birth and they enjoyed a relationship-based approach to care. However, the managerial role of the midwives sometimes made it difficult for them to uphold their ideals, oftentimes leading to frustration and stress of conscience. While some midwives feared that supporting normal birth was at risk of becoming a lost art due to increased medical interventions, there were others who managed to strike a balance between the needs of the organisation and those of the birthing women.

#### The emotional labour of supporting women in labour

The midwives described birth as natural and potentially empowering. Some of them viewed the ideal birth as physiological without synthetic oxytocin or epidural analgesia. These midwives saw labour pain as constructive, positive, and as part of the preparation for and transition to parenthood.

*‘Birth is one of the last natural and primal things we have left… we are mammals… it’s incredibly powerful… one of the few things we can’t control*,* just natural*,* bodily things. I like to deliver women who don’t have any* [pharmacological] *pain relief… you can always use labour pain* [as a guide]. *The pain is what brings out that primal force that empowers women.’* ~Midwife 2~.

Aside from the birth of a healthy baby, becoming involved in the couple in a relationship based on reciprocity and equality was seen as the most rewarding part of the work and the midwives described feelings of elation and euphoria associated with partaking in the birth of a new baby and a new family.

*‘I think*,* well of course*,* besides being present when a new life enters the world*,* it’s like… when there’s a connection with the parents. Like*,* when you’re having a great time*,* everything is running smoothly*,* and you notice that they appreciate what I have to offer. Just that feeling you get when our personalities match… it gives me a huge kick… that they appreciate what I am doing.’* ~Midwife 6~.

A prerequisite for building these relationships was the midwife’s ability to read the room, using her intuition as a tool during labour and birth. The midwives described feeling others’ emotions and energies and interpreting the birthing woman’s non-verbal cues as a natural and fun part of the job. Although described as rewarding, the emotional labour of caring for birthing women, building trusting relationships under exceptional circumstances, and investing so much of themselves in work while remaining professional, took its toll on the midwives.

*‘That’s why you’re exhausted at times*,* it’s amazingly challenging. You establish this incredibly intense and intimate relationship with the couple*,* in just one work shift. You must use all your social skills*,* even if you’re having a bad day. Well*,* actually*,* we can’t have a bad day. Or then we must find a way to hide it.’* ~Midwife 2~.

#### Multitasking midwives balancing birthing women’s needs and organisational demands

Apart from assisting women during labour and birth, the midwives had many other tasks and were rarely seen sitting down, even in times of light workload.

*‘When not occupied in a birth room assisting a birthing woman*,* the midwives provide phone consultations*,* perform check-ups on women coming to the labour ward for assessment*,* care for women admitted to the hospital with complications during pregnancy*,* consult physicians*,* document care*,* interpret cardiotocographs*,* and assist colleagues at the postnatal and gynaecological wards. They are constantly on the move with the ward phone in their pocket*,* going in and out of the birth room*,* to the midwives’ station to check on colleagues*,* into another birth room*,* and back again. When there is no woman to tend to*,* they use their time to tidy up the midwives’ station and the pharmacy room*,* ordering medications or other medical supplies*,* ensuring the labour ward is always in perfect condition.’* ~Observations from Sites I and II~.

The accumulation of these many responsibilities may have contributed to the fact that when in the birth room, midwives sometimes became task-oriented and more focused on ‘doing’ than on ‘being’– inserting intravenous lines, managing infusion pumps, performing vaginal exams or amniotomies, administering drugs and injections, or focusing on the fetal monitor. The partogram, used at both sites to monitor labour progress and ‘achieving’ cervical dilation at a specific rate, may also have influenced the midwives’ work pace, and labour augmentation with synthetic oxytocin was common. Taken together, the managerial role of the midwives contributed to increased intrapartum inventions, added to occupational stress, frustration, and feelings of inadequacy for the midwives and limited women’s access to continuous support.

*‘The woman is quiet and seems emotionally shut down. The midwife interprets her silence as wanting to be left alone. The labour is very quick– the woman’s cervix has dilated 2 cm in 10 minutes. Although the midwife has no other woman to tend to*,* she goes in and out of the room*,* performs vaginal exams or increases the drip speed. During one contraction*,* the baby drops from the ischial spine to crowning*,* and the midwife almost misses catching it.’* ~Observation 3~.

In the same vein, multitasking was highly valued, and one midwife proudly told of one work shift where she caught three babies before lunch.

*‘I delivered three women in a short time and by two o’clock in the afternoon I was done with everything*,* suturing*,* documentation*,* and all. My colleagues were extremely impressed and as I was quite new as a midwife then*,* I really enjoyed it because it was a way to prove myself worthy.’* ~Midwife 2~.

#### Midwifery at risk of becoming a lost art

One midwife expressed concerns about physiological birth and the midwifery skills needed to facilitate it being lost due to an increasingly interventionist labour and birth care.

*‘We’re there to support. Midwifery as a profession started with supporting women in their homes and assisting them in getting the baby out. As birth was hospitalised*,* and with the involvement of medicine*,* so much has happened that has removed the focus from the woman*,* and birth became… I don’t know… it’s like it’s not the woman and the woman’s body who are doing the job anymore*,* it’s like it’s the midwife having the baby. That’s how it feels. I think we stopped appreciating and valuing natural birth. It’s not that natural anymore… today*,* midwives must fight for their professional competencies and to be able to work in the way midwives always have*,* without having to resort to drugs and such.’* ~Midwife 3~.

There was however one example of how a senior midwife found a balance between ‘high-tech’ and ‘high-touch’. By combining her medical knowledge and technological skills with her experiential and intuitive/tacit knowledge, she was able to support physiological birth and intervene, when necessary, not routinely.

*‘The woman*,* giving birth to her first baby*,* has about one contraction every ten minutes. The contractions are spaced out but efficient and labour is progressing well judging by cervical dilation. No epidural. The midwife feels the duration*,* frequency*,* and strength of the contractions by external palpation of the uterus and decides not to augment labour with synthetic oxytocin. This is against the local guidelines*,* where the recommendation is to have three to five contractions every ten minutes. The midwife is aware of nonverbal cues and stays close to the woman*,* breathes with her through the contractions*,* and strokes her gently. When she speaks to the woman*,* she squats or places herself on a low stool to be on the same level as the woman to not make her feel inferior. Her voice and movements are calm*,* she uses simple language and lots of affirmations*,* and she explains what is happening and what she is doing. She guides the woman to trust her body and she also addresses and involves the partner. The midwife monitors the baby’s heart rate intermittently during active labour*,* and continuously during the pushing phase. In a couple of hours*,* a healthy baby is born*,* no vaginal tearing*,* normal blood loss*,* no stress. The new parents are crying tears of joy and relief.’* ~Observation 5~.

### Birthing women trusting in the system

Most women had no special wishes for their births but trusted and relied on the midwife’s expertise to keep her and her baby safe, placing her wants and needs second to others’. Both the birthing women and the midwives presupposed a certain degree of compliance on the woman’s part, which sometimes resulted in unconsented interventions. When trying to make sense of unconsented routine procedures, women tended to blame themselves and their ‘deficient’ bodies. Nevertheless, when evaluating their overall birth experiences, the women were very satisfied, with an average VAS-score value of 8.1 out of 10 (Table 1).

#### Placing the needs of others before their own

Despite the physical and psychological demands of childbirth, the women were very polite and kind during labour and birth, inclined to put the needs of others– the baby’s, the partner’s, the midwife’s, or even other birthing women’s– ahead of their own. Demonstrating the complexity of the balance between women’s autonomy and self-sacrifice in childbirth, one woman– taking into consideration not only her own comfort and wellbeing but also that of her baby, was ready to comply with whatever the midwife suggested. Depending on the outcome of the midwives’ suggestions, this was not necessarily perceived as negative.

*‘As long as everything is ok*,* like there are no complications*,* I think you* [the woman] *can make the decisions. That doesn’t necessarily mean that you’re right. Like for me*,* I had told my partner that the only birth position I wouldn’t try was hands and knees. And then the midwife tells me to try it*,* and I’m like “no way”*,* but she insisted*,* so I didn’t have much choice*,* but when I tried*,* it was great.’* ~Woman 8~.

During the observations and in the interviews, the women were very grateful for the support of their partners. Throughout the births they ensured that the partner was ok, asking if he needed to step outside the birth room for some time or if he needed something to eat. In one observation, one woman apologised continuously to her partner for squeezing his hand too tight during contractions. Another woman was surprised and very grateful that her husband made it through birth, something she had not taken for granted.

*‘He’s so sensitive*,* if he sees blood he will faint*,* but he managed the full day*,* that’s amazing! I didn’t think he would be there the entire day with me.’* ~Woman 3~.

There were examples of women who felt they did not receive adequate pain relief or support because the midwife was busy elsewhere. Nevertheless, they belittled their own needs and were very understanding towards the midwives. One woman made excuses for the midwife’s absence by sympathising both with the midwife for her heavy workload as well as with other birthing women in need of her care.

*‘I think they were quite busy last night*,* even I noticed although I was in excruciating pain. But I mean*,* there was no harm done*,* the contractions were terrible*,* but I understood that there were women who were far worse off than me*,* women who really needed help… But we were ok*,* I understand that you must prioritise*,* and in a way*,* I felt that if they’re not prioritising me*,* I’m probably doing fine somehow*,* which I found reassuring.’* ~Woman 1~.

#### Compliance presupposed

When preparing for birth, most of the first-time mothers opted for a natural birth, read books, searched the Internet, and asked friends and relatives about their experiences. The women who had given birth previously drew on their past experiences. Of the eight women, only one had written a birth plan while the others decided to go with the flow, open to labour and birth not turning out the way they expected or wanted it to.

*‘About birth plans… I understand that there’s less focus on them nowadays*,* to plan how you think or wish your birth to be because*,* in the end*,* it doesn’t turn out like that anyway. So*,* I thought I should go with the flow*,* make decisions in the moment depending on what happens then and there.’* ~Woman 6~.

All women trusted the midwives, viewing them as the ‘birth experts’ knowing better than the women themselves what would benefit them. In one observation, the midwife performed frequent vaginal exams and tried to remove a cervical lip, something that the woman found excruciatingly painful and consequently asked the midwife to stop. When interviewed afterwards, the woman said she felt bad for advocating her needs and thus being a ‘difficult patient’.

*‘I regret that I defied the midwife and said no to the internal examinations… But when I said no*,* she waited*,* and I got ten seconds to breathe… I thought the vaginal exams were worse than the actual contractions… I understand why she did it*,* that she was moving her fingers around to get rid of the cervical lip*,* but it was awful. I would rather have taken five contractions than her touching me like that. It’s not unpleasant*,* it’s pure pain… But at least she listened when I said “Wait”. I mean you’re already vulnerable as it is… I understand that it had to be done*,* but if I ask for ten seconds*,* I must have ten seconds. And I did get that*,* so I was ok. Like that we could meet each other halfway and she got what she wanted*,* and I got what I wanted*,* so then both of us were satisfied*,* I guess.’* ~Woman 1~.

The view that women should compromise their wishes for birth was also shared by the midwives. On the topic of power and compliance in the birth room, one midwife stated:

*’Once they come to the labour ward*,* we have a say in decision-making… home birth*,* that’s a different story*,* but in the hospital*,* they must comply with our rules and routines. That doesn’t mean to ride roughshod over the woman’s wishes*,* but to explain and motivate your actions… So yes*,* I do have power over her*,* quite a lot of power*,* I would say*,* but I hope I use it wisely.’* ~Midwife 4~.

#### Unconsented interventions and self-blame

There were several examples of interventions that were done to, on, or in the woman’s body, without her explicit consent. While interventions at times are necessary for a safe birth, in most cases, they were presented as part of routine care, leaving no room for discussion.

*‘The midwife prepares a pudendal block and without asking the woman if she wants it*,* she injects the anaesthetic during a vaginal exam and says that it is better to have it now so that she will enjoy its full effect when it is time to push. Once in the pushing phase*,* the midwife injects more local anaesthetic into the perineum*,* at the same time telling the woman: “This might sting a little but here’s some more anaesthetic in case I need to give you a cut”.’* ~Observation 7~.

When informed consent was not practised and the midwife failed to motivate her actions, women were sometimes left feeling inept and tried to justify and make sense of the situation by placing blame on, what they felt, their deficient bodies. One woman, who by the midwife was subjected to early and forced pushing lying flat on her back, and who pushed her baby out on sheer willpower, without the aid of spontaneous contractions, wondered if her body was genetically predisposed to experience a complicated pushing phase.

*‘… I don’t know if it has anything to do with it*,* but both my mother and my sister had difficulties pushing their babies out*,* and I felt the same. So I felt a little frustrated over my own incapacity*,* I mean I don’t know why I didn’t feel the urge to push and why I didn’t find the right technique.’* ~Woman 6~.

## Discussion

The aim of this study was to explore the paradox of childbirth becoming increasingly medicalised despite the vast evidence to the benefits of low-intervention physiological birth. We claim that the way that childbirth is organised, ‘managed’, and performed, may be suggestive of societal constructions of gender leading both to gender normative performances and to gender-based hierarchies of power, with obstetricians at the top, midwives in the middle, and birthing women at the bottom.

### The organisation

Drawing on Acker [33], we contend that a society or organisation is not gender neutral but built on norms of masculinity and femininity. This is evident in the division between paid and unpaid work, where women do most of the latter, in income and status inequality between men and women, and in what kind of work qualifies as important. Even though most obstetricians in Sweden today are women, medicine as a profession has historically, by the exclusion of women from higher education, been reserved for men. Regardless of the biological sex of the practitioner, medicine is built upon certain notions of masculinity, such as rationality, hierarchical authority, technical skills, and objectivity, in the same way that nursing (and midwifery) is built on feminine ideals, such as the ability to nurture, give comfort, make emotional contact, and subordination (to medicine) [34]. With time, the boundaries between medicine and nursing/midwifery have been somewhat renegotiated, but our results show that the hierarchy still prevails, where medicine takes precedence over midwifery even though both professions are female dominated in Sweden. Although midwives are responsible for normal birth, doctors are formally responsible for overall medical care, and the ones who make the final decisions. We found that the authoritative power of the doctors led to midwives’ acquiescence, and to interprofessional tension, not always in the best interest of the birthing woman.

In addition to hospital hierarchies, risk discourse and staff shortage were identified as organisational obstacles to promoting physiological birth. The focus on risk in childbirth has previously been explained by (western) society becoming increasingly risk aware during the 20th century, and that health care professionals’ fear of making fatal mistakes and of feelings of guilt, shame, blame, and also of litigation, in combination with a strong orientation towards science, high technology, and efficacy, have propelled a movement towards an interventionist, high-tech birth [9, 14, 35, 36]. The construction of childbirth as risky has also been argued stems from a biomedical view of the male body as the properly functioning prototype (the norm), and the female body as the deviant and defective one, thus in need of surveillance and manipulation to function [35, 37]. Taking history into account, our results, which show that birthing women are closely monitored, and their bodies manipulated through various interventions, may be seen as signs that the legacy of biomedicine and patriarchy still holds strong in contemporary labour and birth care.

Contradictory to the evidence of the benefits of continuous midwifery support to mitigate risk, midwives were due to staff shortage replaced by technological and medical solutions. This may be viewed as an organisation more focused on male coded characteristics like efficacy and productivity, ensuring a constant ‘patient’ flow. While the midwives were highly technically skilled, they were generally less invested in support techniques such as breathing, massage, or encouraging the woman to alternate birth positions. The assumption that the use of technology is an efficient use of midwives’ time and that it decreases their workload may however not hold entirely true. Once the normal physiology of birth has been interfered with, there is the risk that one intervention gives rise to further interventions in a so-called intervention cascade [4, 38]. Apart from subjecting the birthing women to over-intervention, this may in fact lead to more work for the midwives, such as monitoring for the effect of the intervention itself, more check-ups, and more documentation.

### The midwives

In line with Allen [39], describing the organisational elements of nursing work, the midwives were placed in the middle of the hierarchy, below the doctors, and above the birthing women, acting as the organisational glue in the system, and trying to balance organisational demands of efficiency and productivity at the same time as providing continuous support for the birthing women.

Previous research has described the labour ward as a ‘field of tension’ [40] and the ‘cognitive dissonance’ [14] midwives experience ‘doing midwifery between different belief systems’ [41], wanting to promote physiological birth but feeling compelled to ensure institutional throughput, and to hasten birth through various clinical interventions [42]. This is congruent with our findings, where the midwives described the ideal birth as undisturbed and physiological with no or few interventions, but that they for organisational (staff shortage), hierarchical (medical model and physician prerogative), and safety (birth construed as risky) reasons felt hindered to practice midwifery in line with both ideals and evidence.

This may explain the frustration the midwives expressed of being caught between the ideals of physiological births and the reality of a medicalised birth care, as they recognised the value of emotional support but even at times of light workload they became task-oriented and more focused on ‘doing’ than ‘being’, continuously accompanied by feelings of guilt for not helping colleagues if they stayed too long in the birth room. This mirrors the findings of Davis and Homer [43], who showed how midwives disciplined their behaviour according to the unwritten rules of the workplace, being expected and expecting themselves to ‘look busy’ and hence attend to tasks that drew their focus away from the birthing woman.

What is perceived as expectations on one’s behaviour is determined by the social norms in that context, where gender normative performance is one aspect. Martin [44] uses the term ‘internalized technologies of gender’ to describe how individuals, through practices and discourses construct and discipline themselves in line with the dominant notions of gender to the point that it becomes an internalised part of the self. Because of cultural expectations, and because it is an essential part of childbirth support [3], women (here midwives) are expected (and expecting themselves) to be inherently caring, empathising, negotiating, and relational, and providing emotional support is perceived as a ‘natural female’ skill [21, 45]. At the same time, the managerial role and the emotional labour and care work, often performed by mostly women in their homes and at their workplace, have been described as ‘invisible’ work, and culturally and economically devalued [46].

The busy and multitasking midwives in this study may thus be interpreted as a way for them to make their work visible, as action and technological skills are more noticeable and linked to masculinity, and thus more valued, than the invisible emotional care work, here supporting a woman in labour [33]. There is also the need to consider that the midwives, when interviewed, described that even though they enjoyed building trusting relationships and supporting a woman to have a physiological birth, they also considered it hard work, and at times exhausting and ‘amazingly challenging’. This could explain why one midwife said that suggesting an epidural was not only in the interest of the birthing woman but also mitigated some of the negative effects of the midwives’ emotional labour. However, this resulted in occupational stress and stress of conscience for the midwives, when bending over backwards in trying to solve an impossible equation where they are damned if they do and damned if they don’t (provide emotional support/manage workflow through interventions). Ultimately, it resulted in an interventionist labour and birth care, cementing the idea of women’s bodies as flawed by design and in need of intervention for a safe birth.

### The birthing women

Contemporary birth practices must also be viewed from the part of the main characters, namely the birthing women. Women who give birth today were born into an era of technology and have been found to appreciate the technocratic approach to childbirth, as it instils them with a sense of safety and provides them with a relatively pain-free birth [36, 47]. How birth is viewed and culturally understood can also be explained by how it is depicted in media. Chiareli and Beutel [48] found that American movies and television series construct birth as scary, dramatic, or funny, and reinforce gendered behaviours during birth, in displaying birthing women as irrational, ‘crazy’, compliant, and dependent on and rescued by men, whether that be their male partners, friends, or doctors. In contrast, Scandinavian birthing programmes generally depict birth as a natural and potentially empowering event and birthing women as motherly, caring, and communicative [49]. To what extent these depictions influence birthing women’s views on childbirth is beyond the scope of this paper, but they demonstrate how childbirth is socially and culturally constructed, with different representations of femininities simultaneously at play.

We found that women placed the needs of others before their own, that they trusted and relied on the midwife to make just decisions, and that both the birthing women and the midwives expected a certain degree of compliance on the woman’s part, which sometimes led to unconsented interventions. In line with our findings, a recent Swedish study found that less than one in five women consented to interventions during the second stage of labour [50]. Women being ready to compromise their own wishes for birth may be interpreted as them being humble to the unpredictability of childbirth. However, it may also be indicative of normative expressions of femininity and women abiding by a silent contract of compliance, also subscribed to by the midwives.

Gilligan [51] has highlighted how girls and women, through societal ideals of femininity, often put the wants and needs of others ahead of their own, subjected to what she calls the ‘tyranny of nice and kind’, i.e. the societal demand on girls and women to be relational, caring, polite, and selfless towards others, while losing themselves in the process. In the context of childbirth, these normative standards of femininity may prove detrimental to the birthing woman’s agency, causing her to self-discipline into a compliant and apologetic ‘good patient’, socially programmed to gratefully accept whatever is suggested or done to her, thus unwittingly contributing to being placed at the bottom of the hierarchy [44]. However, normative expressions of femininity may also be beneficial in the context of childbirth. Tanassi [52] argues that women use compliance (with a known caregiver) as a strategy, aligning themselves with the obstetric system to achieve ideals of a self-sacrificing ‘good mother’, thus fulfilling societal expectations on motherhood. Although this strategy may momentarily provide women with certain benefits, self-sacrifice is highly questionable in a longer perspective. Furthermore, in women’s evaluations of their birth experiences, they tend to focus more on the midwife’s ‘being’, i.e. the relationship with the midwife and the midwife’s characteristics and caring traits and how she made the woman feel, rather than on her ‘doing’, i.e. performing interventions [53]. This demonstrates that normative expressions of femininity should not be underestimated as a powerful and positive tool during childbirth. The problem is rather how these expressions, like being kind, gentle, and caring, are valued in society and where they are placed in a gender-based hierarchy of power.

Women’s compliance may also be explained by ‘women don’t know what they don’t know’ [54]. Oftentimes, the only birth/s a woman will attend is her own, and the hospital birth room is a secluded space, relatively free from insight. Not underestimating the importance of the care provided by the midwives, indicated by the women’s high VAS-scores for overall birth experience (Table 1), the scores may also be interpreted as women not knowing what to be critical of, but assume ‘what is, must be best’ [55]. This may also explain why some women viewed their bodies as dysfunctional and turned to self-blame when exposed to unconsented interventions.

## Conclusion

We suggest that societal gender norms and gender-based hierarchies, in combination with modern society becoming progressively risk-laden and technology-oriented, have contributed to an increasingly medicalised and interventionist labour and birth care organisation, where physiological birth is rare.

The labour care organisation was hierarchical and based on traditional masculine values such as rationality, efficiency, and productivity. The midwives tried to balance the needs of the birthing women and the organisational demands of throughput, but as action and technological skills are more noticeable and linked to masculinity, and thus more valued than the invisible femininity-coded emotional care work of supporting a woman in labour, the midwives became task-oriented and more focused on ‘doing’ than on ‘being’. In turn, this led to more birth interventions, less continuous support for the birthing women, and to occupational stress and stress of conscience for the midwives. Normative expressions of femininity were observed in the birthing women, such as placing the needs of others before their own and acts of compliance, which sometimes led to unconsented interventions.

Given the many benefits of a woman-centred midwifery care for maternal and neonatal health and well-being, more efforts need to be made to define the many aspects of midwifery care. For this purpose, more attention needs to be drawn to the value of companionship in labour and relationship-based care, which are as vital as clinical assessment in childbirth.

Awareness of how gender norms inform labour and birth care practice may be one way to make visible and to recognise all aspects of midwifery care, as well as help flatten hospital hierarchies, improve working conditions for midwives, limit unnecessary and unconsented interventions for the birthing women, and promote physiological birth.

## Data Availability

The datasets generated and/or analysed during the current study are not publicly available due to their containing information that could compromise the privacy of research participants. However, they are available from the corresponding author upon reasonable request.
